# High-frequency synthetic apomixis by *OsBBM1* shows environmentally sensitive inheritance instability in hybrid rice

**DOI:** 10.3389/fpls.2026.1747393

**Published:** 2026-02-09

**Authors:** Yao Wang, Siqing Liu, Youhui Wan, Jiaying Liu, Qiming Lv, Mengliang Cao, Yumei Xia

**Affiliations:** 1 School of Breeding and Multiplication (Sanya Institute of Breeding and Multiplication), Hainan University, Sanya, China; 2 College of Biology, Hunan University, Changsha, China; 3 State Key Laboratory of Hybrid Rice, Hunan Hybrid Rice Research Center, Changsha, China; 4 National Center of Technology Innovation for Saline-Alkali Tolerant Rice in Sanya, Sanya, China

**Keywords:** apomixis, hybrid rice, *OsBBM1*, temperature-sensitive, unstable inheritance

## Abstract

Synthetic apomixis allows for the fixation of heterosis in rice, eliminating the need for annual hybrid seed production. Recently, synthetic apomixis has been successfully implemented in rice, enabling stable clonal propagation through seeds via a combination of *MiMe* (Mitosis instead of Meiosis) and parthenogenesis/haploid induction systems. However, our previous research also identified unexpected transgenerational inheritance in one apomictic line suggesting additional regulatory mechanisms require further investigation. In this study, we propagated ten apomictic lines exhibiting high clonal seed frequency (T_0_ generation) to T_3_ generation across two geographic locations. Ploidy levels of progeny plants were assessed at each generation. By analyzing progeny clonal seeds frequency across generations and locations, we demonstrated that seven apomictic lines maintained stable clonal progeny, while one progressively declined across generations. Surprisingly, two apomictic lines exhibited alternating high and low induction rates when cultivated in different locations, suggesting transient genetic instability rather than permanent modification. We analyzed temperature variations between the two sites and propose that elevated temperatures during flowering may modulate *OsBBM1* expression or translation, consequently affecting parthenogenesis efficiency. Additionally, transgene silencing may account for the observed progressive decline in induction rates. Our findings have profound implications for both the fundamental biology of synthetic apomixis and its commercial application, highlighting the critical importance of optimizing cultivation environments for stable clonal seed production.

## 1 Introduction

Heterosis, also known as hybrid vigor, is a fundamental biological phenomenon characterized by the enhanced performance of hybrid progeny in traits such as growth vigor, stress tolerance, yield potential, and product quality relative to their parental lines (Chen, 2010; Hochholdinger and Baldauf, 2018). This phenomenon has been extensively exploited in modern crop breeding programs to boost agricultural productivity. However, the Mendelian segregation of genetic traits in subsequent filial generations poses a significant limitation, requiring the recurrent and costly production of F_1_ hybrid seeds to sustain optimal crop performance (Spillane et al., 2004). Apomixis, a form of asexual reproduction through seeds, presents a transformative solution by enabling the fixation of heterosis through clonal propagation of elite F_1_ genotypes (Koltunow and Grossniklaus, 2003). This innovative approach not only circumvents the economic constraints associated with annual hybridization but also facilitates the large-scale commercialization of genetically stable, high-performance hybrid crops (Mahlandt et al., 2023).

The development of synthetic apomixis in rice has witnessed remarkable progress in recent years, offering unprecedented opportunities for fixing hybrid vigor in elite cultivars. Current apomictic strategies primarily exploit two fundamental mechanisms: parthenogenesis and haploid induction systems. The field achieved its first major milestone when Khanday et al. (2019) successfully generated apomictic rice lines demonstrating a 29.2% clonal seed formation rate through synergistic combination of the *MiMe* (Mitosis instead of Meiosis) system with ectopic expression of the embryogenesis regulator *OsBBM1* in egg cells (Khanday et al., 2019). Subsequent advances in genome editing technology enabled Wang Kejian’s team to create apomictic hybrid rice with 5.23% clonal seed induction efficiency via simultaneous knockout of four meiotic genes (*REC8*, *PAIR1*, *OSD1* and *MTL1*) (Wang et al., 2019). A groundbreaking improvement came with Vernet et al.’s work, which achieved >95% clonal seed induction by precisely regulating *OsBBM1* expression using the egg cell-specific *OsECA1* promoter in conjunction with the *MiMe* system (Vernet et al., 2022). The genetic toolbox for apomixis induction has since expanded considerably, with multiple alternative approaches demonstrating efficacy, including *MiMe* combination with various embryogenesis factors (*OsBBM4* (Wei et al., 2023), *ToPAR* (Song et al., 2024), *OsWUS* (Huang et al., 2025), *PpPAR* (Xiong et al., 2025)), supplementation with *AZP2* (Song et al., 2024), or targeted mutation of *OsPLDα2* (Hu et al., 2025).

Recent studies have provided compelling evidence for the generational stability of engineered apomixis in rice. Vernet et al. systematically demonstrated that multiple apomictic lines maintained consistently high clonal seed induction rates across T_1_-T_3_ generations while preserving stable agronomic performance and heterozygosity patterns (Vernet et al., 2022). Similarly, Liu et al. reported stable maintenance of key characteristics - including agronomic traits, induction efficiency, genomic integrity, and methylation profiles - from T_1_ to T_4_ generations in materials developed through quadruple knockout of *REC8*, *PAIR1*, *OSD1* and *MTL1* genes (Liu et al., 2023). These findings were further corroborated by Song et al., who observed stable transgenerational inheritance of clonal seed induction rates, seed setting rates, and heterozygosity in T_1_-T_3_ generations of apomictic materials (Song et al., 2024). Collectively, these studies establish that synthetic apomixis can reliably produce materials with either high induction rates or high seed setting rates, while consistently maintaining three crucial intergenerational stability parameters: (1) clonal seed induction efficiency, (2) agronomic performance, and (3) genetic/epigenetic integrity. This multi-generational stability represents a critical milestone for the practical application of apomixis technology in crop improvement programs.

Building upon our prior success in generating apomictic rice materials through transformation of Yongyou 4949 with vectors p94C (*‘sgMiMe’_pAtDD45:BBM1*) and p95C (*‘sgMiMe’_pAtMYB98_pAtDD1_pOsECA1-like1:WUS_pAtDD45:BBM1*) (Dan et al., 2024), we observed that while most lines maintained stable clonal seed induction rates across generations, some exhibited transgenerational inheritance variations. To address this, we conducted concurrent multi-generation and multi-site evaluations of high-performance lines (five p94C and five p95C lines with >80% induction rates) enabled comprehensive characterization of genetic stability and preliminary elucidation of instability mechanisms. These findings significantly advance both theoretical understanding and practical implementation of synthetic apomixis in crop improvement.

## 2 Materials and methods

### 2.1 Plant materials and growth conditions

The experimental recipient material was Yongyou 4949, a 120-day growth cycle *indica-japonica* hybrid rice, as the transformation recipient. All transformed lines, including the ten lines of p94C and p95C, from T_0_ to T_3_ generations, were planted in experimental plots in transgenic field trial sites in Sanya, Hainan, and Changsha, Hunan, and were managed uniformly according to standard field protocols.

For the temperature treatment experiments conducted in Changsha, three plants each of the p94C stable line (HW7), p94C unstable line (HW14), p95C stable line (CL10), and p95C unstable line (CL6) were selected as experimental materials. At the initial heading stage, the plants were transferred into two artificial climate chambers and cultivated until maturity for seed harvest. One chamber was set at a daytime temperature of 35°C and a nighttime temperature of 30°C, while the other was set at a daytime temperature of 28°C and a nighttime temperature of 24°C. Both chambers maintained a photoperiod of 12-hour light/12-hour dark and a relative humidity of 80%.

### 2.2 Plasmid construction and genetic transformation

The two expression cassettes, (*‘sgMiMe’_pAtDD45:BBM1*) and (*‘sgMiMe’_pAtMYB98_pAtDD1_pOsECA1-like1:WUS_pAtDD45:BBM1*), were individually constructed into backbone vectors and designated as p94C and p95C, respectively (Dan et al., 2024). Genetic transformation was performed according to our previously described protocol (Xia et al., 2019).

### 2.3 Transgenic plant identification

Molecular detection was performed on T_0_-generation lines to identify transgenic positive materials for subsequent experimental studies. Genomic DNA was extracted from leaves using the CTAB method (Allen et al., 2006). Positive transgenic lines were initially identified by PCR amplification of *OsBBM1*, followed by sequencing analysis of *OsOSD1*, *PAIR1*, and *OsREC8* to characterize target mutations.

### 2.4 Flow cytometry

Samples were prepared according to the manufacturer’s protocol of the CyStain™ PI Absolute P kit, with DNA content quantification performed using a flow cytometer (FongCyte C2060) to determine plant ploidy levels. In this experiment, we selected early-stage rice leaves for flow cytometric ploidy analysis. The x-axis represents the fluorescence intensity (or DNA content), while the y-axis represents the count (or cell number). The ploidy level was determined based on the peak position (or value) on the x-axis.

### 2.5 Seed setting rate analysis

All experimental materials were cultivated at transgenic research bases in Changsha, Hunan and Sanya, Hainan until full maturity, followed by comprehensive evaluation of both induction efficiency and seed setting rates. Within the planting plots of each line across different generations, 4–5 individual plants were selected for seed harvesting after excluding border rows, followed by seed threshing. The seed setting data were subjected to Duncan’s multiple range test (significance level set at p<0.05) using SPSS software for statistical analysis.

### 2.6 Reverse transcription quantitative polymerase chain reaction

Total RNA was isolated using TRIzol reagent (Invitrogen). First-strand cDNA was synthesized with a reverse transcription kit. Quantitative analysis was performed by RT-qPCR using the synthesized cDNA as the template. Real-time PCR was conducted on a Roche Light Cycler 480 II detection system using SYBR Green dye, with three technical replicates per experiment. The reaction mixture consisted of 5 μL of SYBR Green Mix, 1 μL of cDNA template, 0.2 μL each of forward and reverse primers, and 3.6 μL of ddH_2_O. The amplification protocol was as follows: initial denaturation at 95°C for 30 s, followed by 40 cycles of denaturation at 95°C for 5 s and annealing/extension at 60°C for 30 s. Melting curve analysis and cooling steps were performed according to the instrument’s default settings.

### 2.7 Phenotypic imaging

All plants were carefully uprooted from the field and placed in containers for standardized imaging against a black background using a Canon EOS 5D MarkII digital camera with the following specifications.

## 3 Results

### 3.1 Generational skipping of diploid traits in unstable apomictic lines

**Figure 1:**
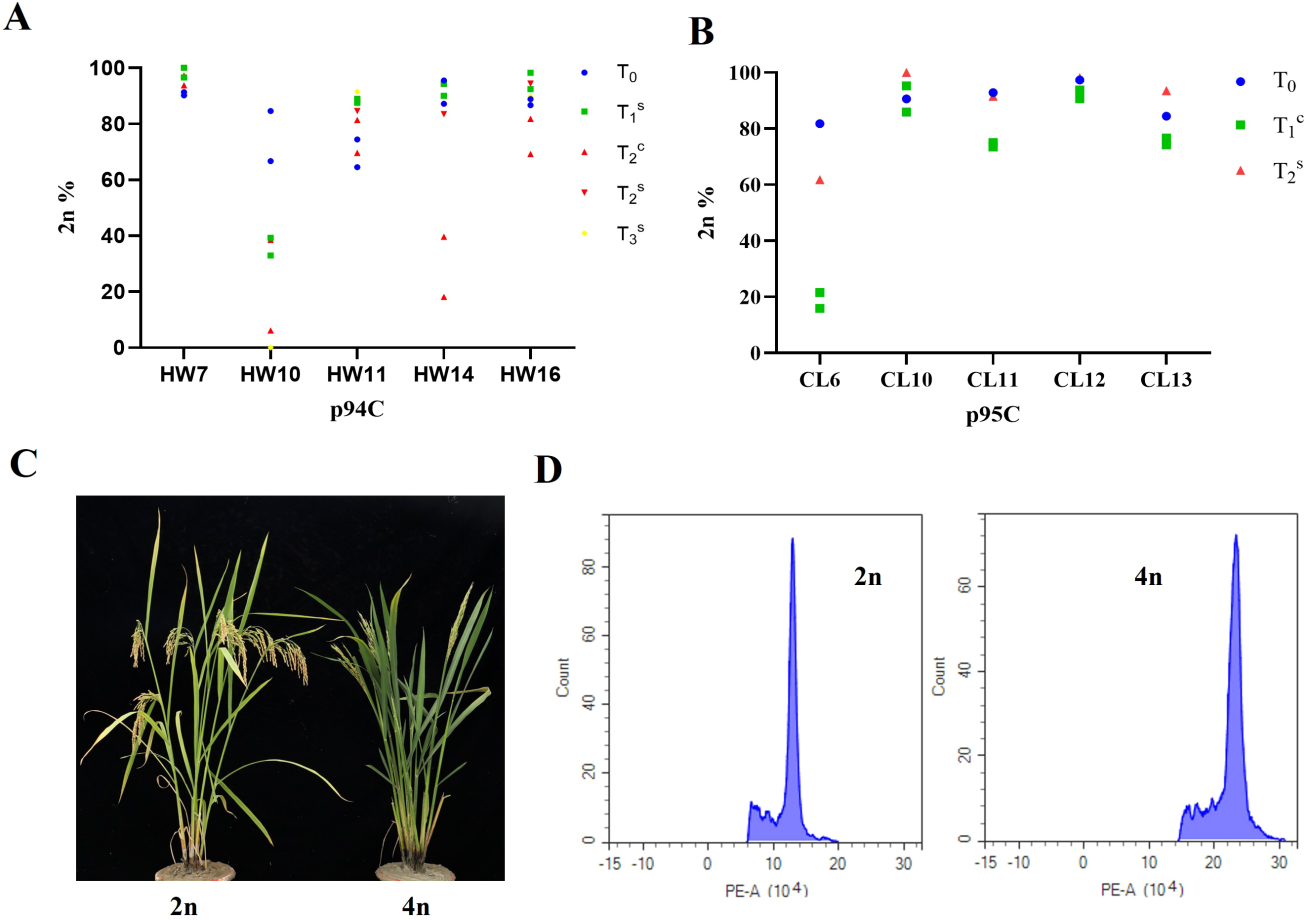
Analysis of clonal diploid plants and phenotypic characterization of apomictic materials cultivated. **(A)** Diploid induction rates of p94C apomictic lines across T_0_-T_3_ generations (x-axis: tested lines; y-axis: diploid induction rate), **^s^** were plants grown at the Sanya transgenic base in Hainan; **^c^** were plants grown at Changsha transgenic base in Hunan, blue circle indicates T_0_ generation, green square represents T_1_^s^ generation, red triangle means T_2_^c^ generation, red diamond denotes T_2_^s^ generation, yellow triangle means T_3_^s^ generation. **(B)** Diploid induction rates of p95C apomictic lines in T_0_-T_2_ generations (x-axis: tested lines; y-axis: diploid induction rate), blue circle indicates T_0_ generation, green square represents T_1_^c^ generation, red triangle means T_2_^s^ generation **(C)** Morphological comparison between diploid and tetraploid plants, showing complete sterility and elongated-awned seeds in tetraploids, left: diploid; right: tetraploid. **(D)** Flow cytometry ploidy analysis (x-axis: fluorescence intensity; y-axis: cell count), left: diploid; right: tetraploid.

In our previous study, both line HW10 (p94C) and line CL6 (p95C) exhibited a decline in clonal seed induction rates across successive generations (Dan et al., 2024). To determine if the observed atavism was stochastic, we cultivated progeny seedlings from five lines each of p94C and p95C of different generations (T_0_, T_1_, and T_2_) in Sanya during winter. We determined the ploidy level of the progeny seedlings of different generations using flow cytometry and identified phenotype (**Figures 1C, D**). The results showed that three lines from p94C (HW7, HW11, and HW16) and four lines from p95C (CL10, CL11, CL12, and CL13) maintained relatively stable diploid induction rates across three generations (**Figure 1A**; **Supplementary Table 1**). However, line HW10 from p94C showed a progressive decline in induction rates across generations at 84.62%, 32.95%, and 6.16%. HW14 maintained stable induction rates in T_0_ and T_1_ (87.18% and 90.08%, respectively), but dropped sharply to 18.14% in T_2_. Meanwhile, line CL6 from p95C exhibited a dramatic decrease in induction rate from 80% in T_0_ (Dan et al., 2024) to 15.87% in T_1_ (**Figure 1A**; **Supplementary Table 1**).In this study, the T_1_ generation (progeny of T_0_) grown at the Sanya transgenic base in Hainan is designated as T_1_^s^, while the T_1_ generation (progeny of T_0_) grown at the Changsha transgenic base in Hunan, China, is designated as T_1_^c^.

To evaluate the impact of cultivation location on diploid plant induction in apomictic materials, we determined the ploidy levels of materials from different generations grown at distinct sites. Among the five p94C-derived apomictic lines, lines HW7, HW11, and HW16 maintained consistently high apomixis induction rates across all four generations (**Figure 1A**). In contrast, HW10 and HW14 showed progressive generational decline (**Figure 1A**). In line HW10, we identified 16, 22, 20, 10, 0 diploid progeny out of 24, 56, 52, 43, 56 individuals that from T_0_, T_1_^s^, T_2_^c^, T_2_^s^ and T_3_^s^ generation, with 66.67, 39.29, 38.46, 23.26 and 0% diploid rates, respectively (**Figure 1D**, **Supplementary Table 2**). Meanwhile, line HW14 showed high diploid rates in T_0_, T_1_^s^, T_2_^s^, and T_3_^s^ generations but experienced a dramatic drop in T_2_^c^, we identified 84, 33, 19, 56, 41 diploid progeny out of 88, 35, 48, 67, 47 individuals that from T_0_, T_1_^s^, T_2_^c^, T_2_^s^ and T_3_^s^ generation, with 95.45, 94.29, 39.58, 83.58 and 87.23% clonal seeds rates, respectively (**Supplementary Table 2**). Among the five apomictic lines derived from construct p95C, line CL6 initially exhibited a high clonal seeds rate (81.82%; 36/44), but showed a sharp decline to 21.57% (11/51) in T_1_^c^, before rebounding to 61.82% (34/55) in T_2_^s^ (**Figure 1B**; **Supplementary Table 2**). The other four lines remained stable across these generations (**Figure 1B**). These results mirrored those from Sanya: HW10, HW14 and CL6 exhibited unstable inheritance patterns, displaying atavism (transgenerational skipping). Concurrently, generations showing decreased induction rates were exclusively associated with Changsha cultivation. Crucially, apomictic materials preserved clonal morphological integrity across generations (**Supplementary Figures 1C–E**), in stark contrast to the segregating phenotypes of self-pollinated F_2_ hybrids (**Supplementary Figure 1B**). In conclusion, the variation in induction rates of unstable apomictic materials is associated with cultivation location. Induction rates decreased significantly when cultivated in Changsha, whereas high induction levels were restored under Sanya growing conditions.

### 3.2 Environmental factors impact synthetic apomictic trait stability

Our multi-location, multi-generational cultivation study in Sanya (Hainan) and Changsha (Hunan) revealed important patterns in apomictic stability. While most materials maintained consistently high induction rates across generations, specific lines displayed notable instability. In line HW10, clonal seed induction rates progressively decreased across generations, indicating persistent and irreversible instability. For lines HW14 and CL6, induction rates varied with cultivation site, suggesting transient genetic instability rather than permanent modification.

**Figure 2:**
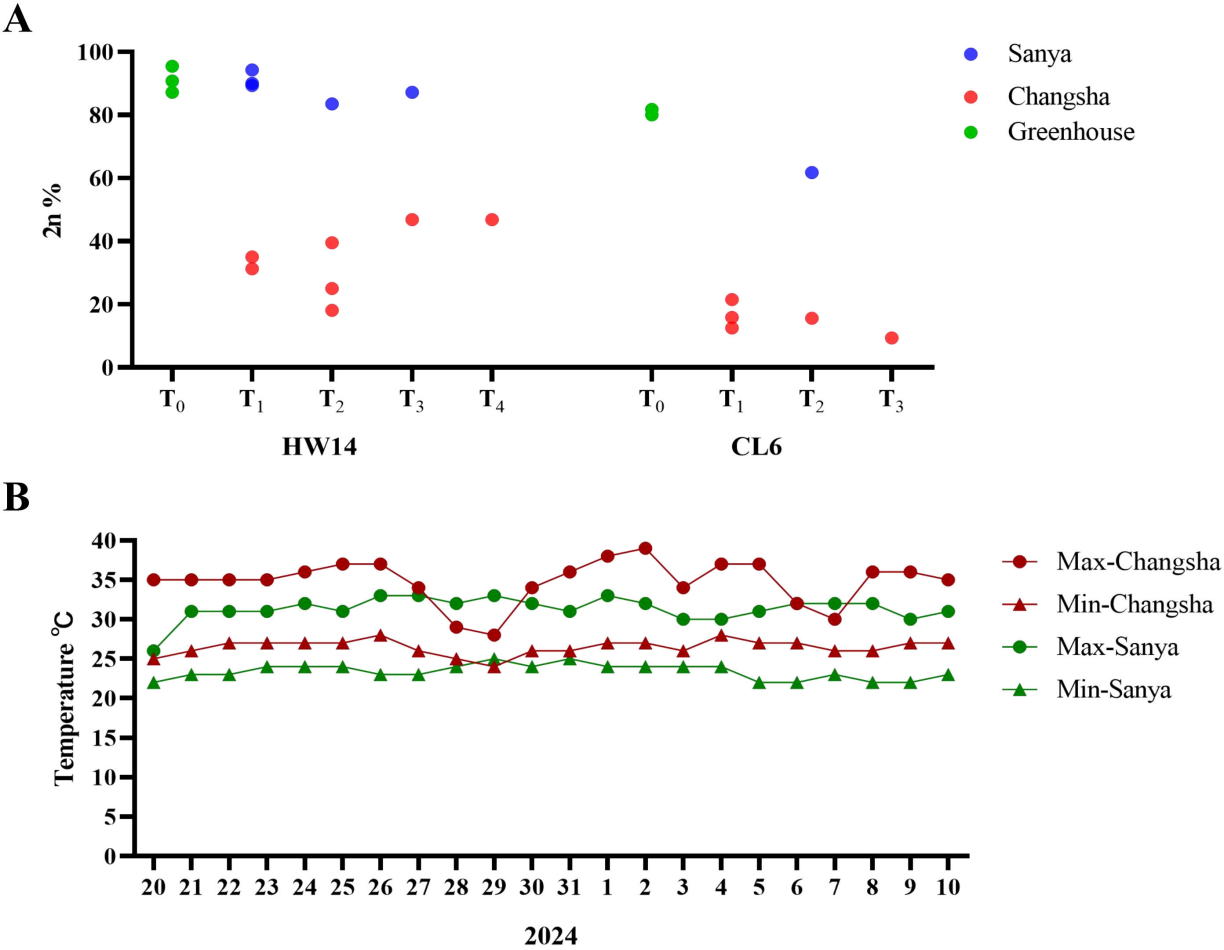
Variation patterns of induction rates in unstable apomictic materials. **(A)** Variation trends in diploid induction rates for unstable apomictic lines HW14 and CL6 (x-axis: different generations; y-axis: diploid induction rate). Blue circles represent cultivated in Sanya, red circles indicate cultivated in Changsha, while green circles means cultivated in greenhouse. **(B)** Temperature profiles during flowering periods in Sanya (March 20 - April 10, 2024), Hainan and Changsha (August 20 - September 10, 2024), Hunan (x-axis: dates; y-axis: temperature). Red indicates Changsha, green means Sanya, the triangle symbols symbolize the daily minimum temperature, circles represent the daily maximum temperature (Source: https://lishi.tianqi.com/).

For the two lines, HW14 and CL6, which exhibited fluctuating clonal seed induction rates, we conducted a further pedigree analysis of their entire planting history. After the T_0_ generation of HW14 and CL6 was cultivated and harvested in the greenhouse in Changsha, the clonal seed induction rates of their progeny ranged from 87.18% to 95.45%. The progeny of the T_1_ generation harvested in Sanya, were cultivated and assessed in both Sanya, and Changsha, yielding clonal seed induction rates of 89.39% and 93.62%, respectively. Conversely, the T_1_ generation harvested Changsha, was cultivated and assessed in the same two locations, resulting in induction rates of 35% and 31.25%, respectively. The progeny of the T_2_ generation harvested in Sanya, exhibited a clonal seed induction rate of 83.58%, while that harvested in Changsha, showed rates ranging from 18.14% to 39.58%. Meanwhile, the progeny of the T_3_ generation harvested in Sanya achieved an induction rate of 87.23%, whereas, the T_3_ generation cultivated and harvested in Changsha yielding only 46.88%. Similarly, for line CL6, seeds harvested from T_0_ generation, yielding induction rates of 80% and 81.82%. The T_1_ generation was cultivated three times in Changsha. Subsequently, the T_2_ generation was cultivated once each in Sanya, and Changsha, and the T_3_ generation was cultivated once in Changsha. All data consistently show that within the same generation, clonal seed induction rates for plants cultivated in Changsha were significantly lower compared to those cultivated in Sanya (**Figure 2A**).

These results establish that while apomictic induction can remain stable across generations under certain conditions (Sanya winter), summer cultivation in Changsha consistently induces a transgenerational suppression of apomictic capacity. This striking location-dependent phenomenon suggests that distinct factors - potentially including temperature regimes (Changsha summer average 28.7°C *vs* Sanya winter 22.3°C) (**Figure 2B**), or photoperiod variations - may epigenetically regulate the heritable expression of engineered apomictic traits. The reproducible nature of this pattern across two independent transgenic lines strongly implies that the observed instability represents an environmentally modulated, potentially reversible epigenetic phenomenon rather than permanent genetic change. These findings have profound implications for both the fundamental biology of synthetic apomixis and its commercial application, highlighting the critical importance of optimizing cultivation environments for stable clonal seed production.

### 3.3 Apomictic materials are susceptible to temperature fluctuations

**Figure 3:**
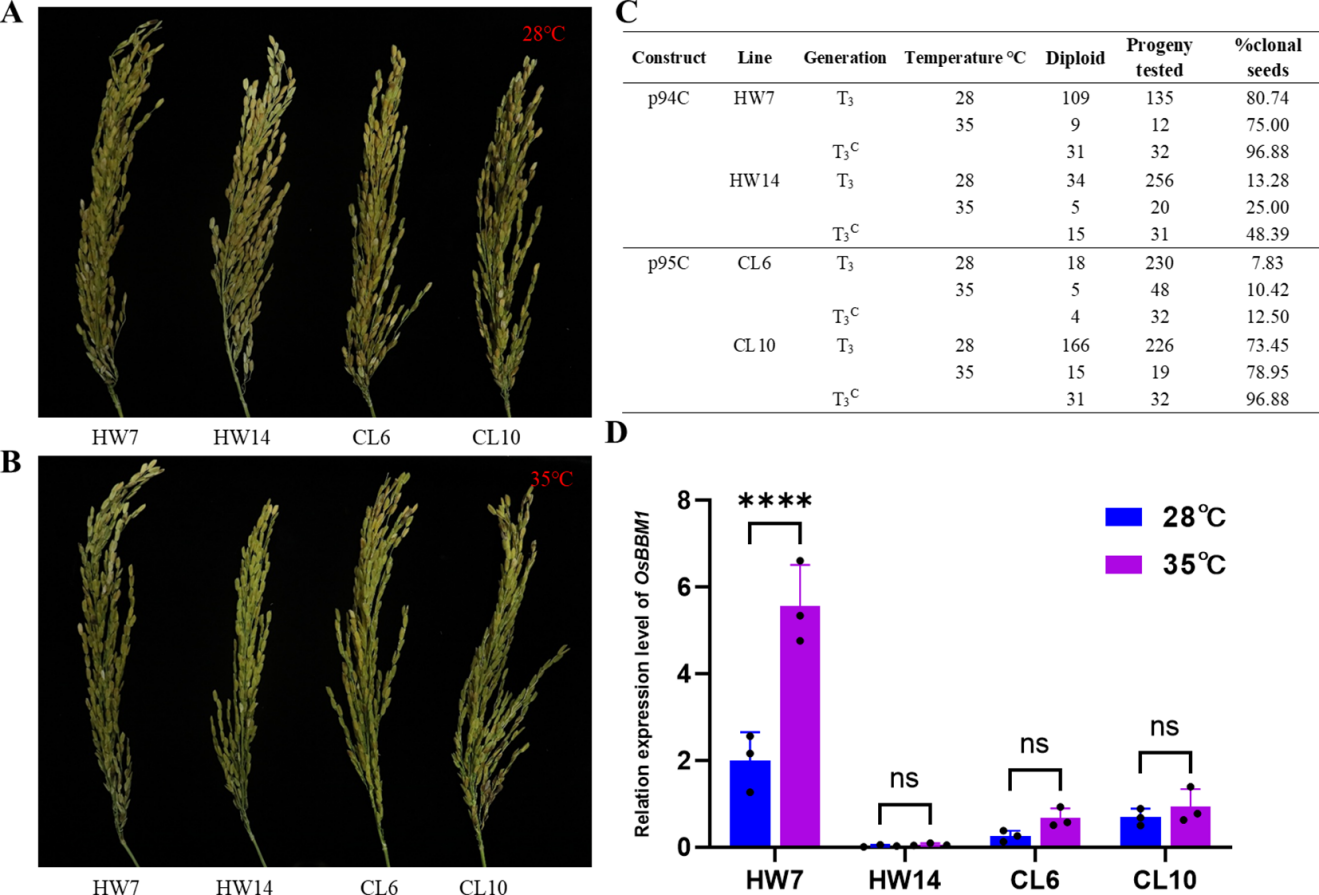
Analysis of fertility, induction and expression under different temperatures in Changsha. **(A, B)** Sample-specific panicle fertility under varied temperature treatments. A: Seed-setting performance of spikelets from HW7, HW14, CL6, and CL10 under treatment in a 28°C artificial climate chamber. B: Seed-setting performance of spikelets from HW7, HW14, CL6, and CL10 under treatment in a 35°C artificial climate chamber; **(C)** Statistical analysis of induction rates for HW7, HW14, CL6, and CL10 after treatment at 28°C and 35°C. **(D)***OsBBM1* expression levels after different temperature treatments in Changsha, n=3. The error bars represent the standard deviation. “****” indicates a p-value < 0.0001, and “ns” indicates no significant difference (x-axis: tested lines; y-axis: expression levels).

After harvesting seeds from materials treated at different temperatures, it was found that for the four lines p94C and p95C, regardless of whether they were stably or unstably inherited, the seed setting rate after 28°C treatment was significantly higher than that after 35°C treatment (**Figures 3A, B**; **Supplementary Table 5**). Subsequently, flow cytometry ploidy identification was performed on seeds harvested from these different temperature treatments after germination induction. The results showed that compared with field conditions in Hainan, the induction rates of the four lines after both 28°C and 35°C treatments decreased. The stable hereditary line p94C (HW7) exhibited induction rates of 80.74% and 75.00%, while p95C (CL10) showed rates of 73.45% and 78.95%, respectively (**Figure 3C**). In contrast, the unstable hereditary lines demonstrated induction rates as follows: p94C (HW14) at 13.28% and 25.00%, and p95C (CL6) at 7.83% and 10.42%, respectively (**Figure 3C**).

To analyze *OsBBM1* expression under different temperature treatments, we collected ovaries from florets 0–3 hours before anthesis for RT-qPCR analysis. The results showed that *OsBBM1* expression levels were higher after 35°C treatment compared to 28°C treatment in both stable (HW7, CL10) and unstable (HW14, CL6) lines of p94C and p95C, indicating that *OsBBM1* expression is temperature-responsive (**Figure 3D**). In conclusion, the greater temperature sensitivity of unstable apomictic heredity is evidenced by its effect on *OsBBM1* expression levels.

### 3.4 Seed-setting rate maintained relatively stable across generations

**Figure 4:**
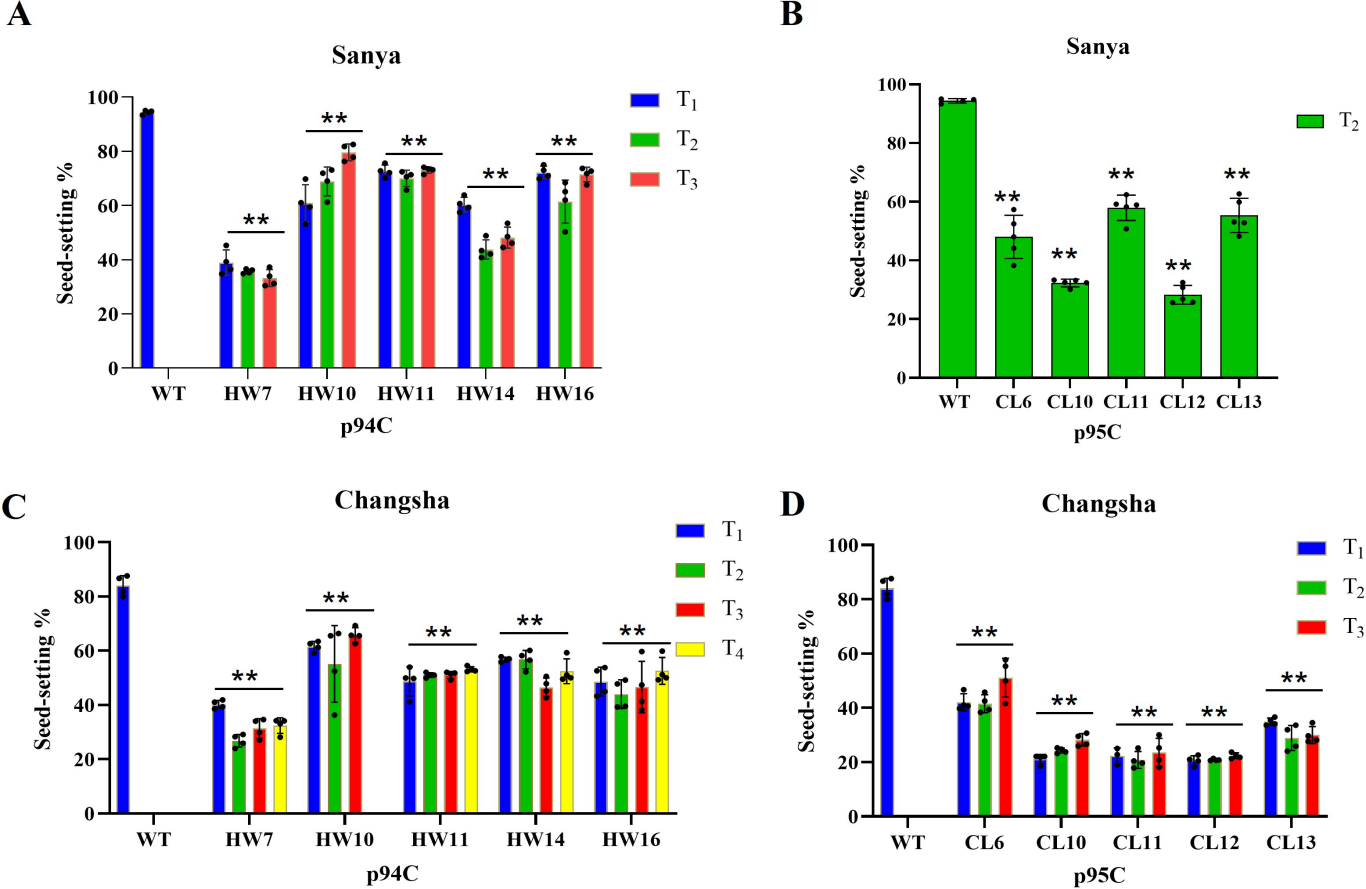
Analysis of seed-setting rate of apomictic materials cultivated in Sanya and Changsha. **(A, B)** Seed-setting rates of apomictic lines across generations cultivated in Sanya (x-axis: tested lines; y-axis: seed-setting rate). The error bars represent the standard deviation. ** indicates a p-value < 0.01, compared with the WT group. In figure A, the sample size for each group is 4. In figure B, the sample sizes for groups CL6, CL10, CL11, CL12, and CL13 are 5. Blue indicates T_1_ generation, green represents T_2_ generation, and red denotes T_3_ generation. **(C, D)** Seed-setting rates of apomictic lines across generations cultivated in Changsha (x-axis: tested lines; y-axis: seed-setting rate). blue indicates T_1_ generation; green represents T_2_ generation; red means T_3_ generation; yellow means T_4_ generation. The error bars represent the standard deviation. ** indicates a p-value < 0.01, compared with the WT group. The sample size for all groups is 4.

Apomictic materials preserved clonal morphological integrity across generations (**Supplementary Figures 1A, C–E**), in stark contrast to the segregating phenotypes of self-pollinated F2 hybrids (**Supplementary Figure 1B**). Ten apomictic lines cultivated in Sanya were recorded seed-settling rates by sampling individual plants across generations (4–5 plants per line). All lines exhibited significantly reduced seed-setting rates compared to the wild-type control (94.39%). The highest rate occurred in the T3 generation of HW10 (79.62%), which showed a progressive increase across generations (60.84%→ 68.84% → 79.62%). Line HW11 demonstrated relatively stable seed-setting rates (72.66%, 70.03%, 73.10%) and stable induction rates ranging from 74.46% to 81.36% across generations. In contrast, line HW7 maintained high and stable induction rates (90.23% to 100%) but consistently low seed-setting rates (38.96%, 35.73%, 33.24%) (**Figures 4A, B**; **Supplementary Table 3**).

Replication trials in Changsha confirmed significantly reduced seed-setting rates versus the wild-type control (84.05%) and seed-setting rates were consistently lower rates compared to Sanya. The highest rate in Changsha remained with HW10-T3 (65.79%). HW11 displayed favorable comprehensive traits, with stable seed-setting rates (48.73%, 51.03%, 51.10%, 53.15%). HW7, despite high induction stability, maintained the lowest seed-setting rates (26.81-40.22%). Apomictic lines of p95C generally underperformed those of p94C in seed-setting, CL6 yielding the highest seed-setting rates of 42.07%, 41.52%, and 51.16% (**Figures 4C, D**; **Supplementary Table 4**). In summary, apomictic lines showed reduced seed-setting rates versus controls, lines exhibiting stable induction rates across generations also demonstrated relatively greater stability in seed-setting rates. This pattern enables the identification of lines with relatively high performance in both traits, exemplified by HW11 in Sanya.

## 4 Discussion

Our research has systematically advanced the development of synthetic apomixis systems for hybrid rice through successive engineering improvements, beginning with the p94C construct combining *MiMe* with egg cell-specific *OsBBM1* expression and subsequently enhancing this system through p95C incorporating *WUS* expression (Dan et al., 2024). Extensive multi-environment evaluation revealed two distinct instability patterns: geographically-sensitive fluctuations in lines HW14 and CL6 directly correlated with cultivation site rotation, progressive T_3_ silencing in line HW10 suggesting transgene inactivation, and contrasting stable high-performance lines, collectively demonstrating that while synthetic apomixis can be achieved, its reliable implementation requires overcoming both environmentally-induced epigenetic modulation and inherent transgene silencing mechanisms - findings that critically inform both fundamental research directions and practical deployment strategies for this transformative agricultural technology.

This study employed Yongyou 4949, a 120-day growth cycle indica-japonica hybrid rice, as the transformation recipient, with distinct flowering periods between test locations: March 20–31 in Sanya, Hainan (average daily max 29-31°C, min 23-25°C in 2023; max 26-33°C, min 22-25°C in 2024) versus August 20–31 in Changsha, Hunan (max 28-36°C, min 22-27°C in 2023; max 29-37°C, min 24-28°C in 2024) (Source: https://lishi.tianqi.com/). Meteorological analysis revealed Changsha’s flowering period exhibited both significantly higher peak temperatures (3-6°C greater than Sanya) and presented significant fluctuations (**Figure 4B**). The enzymatic machinery governing transgene expression (eg., RNA polymerases, ribosomes) exhibits temperature-dependent activity, where elevated temperatures may accelerate mRNA degradation or induce splicing errors, ultimately reducing functional protein yields. This is exemplified by the thermolabile nature of *WUS* mRNA, a key stem cell regulator whose translational efficiency diminishes under heat stress due to structural instability. In Arabidopsis, *WUS* mRNA stability relies on 5’ m7G capping mediated by the MYB3R-like/RID2 (ROOT INITIATION DEFECTIVE 2) complex, wherein heat stress inhibits this capping activity, leading to rapid exonuclease-mediated decay of uncapped transcripts (Liu et al., 2024). Similarly, translational regulators demonstrate thermal sensitivity, as observed in rice where the guanylyltransferase AET1 orchestrates *OsARF* mRNA (e.g., OsARF19/23) translation efficiency via uORF modulation through interactions with ER-localized RACK1A and eIF3h. Heat stress disrupts AET1-OsARF mRNA binding patterns, concurrently impairing both translational efficiency and mRNA stability through secondary structure alterations (Chen et al., 2019). In our study, lines HW14 and CL6 exhibited instability in apomictic induction rates when cultivated under Changsha (higher temperature) and Sanya (optimal temperature) conditions. Subsequent analysis of induction rates after 28°C and 35°C treatments revealed that unstable lines showed greater sensitivity to temperature. Further quantification of *OsBBM1* expression levels demonstrated progressively higher expression with increasing temperature. However, we observed that the induction rate after 35°C treatment did not decrease compared to that after 28°C treatment, which contradicts our earlier hypothesis that high temperature reduces apomixis efficiency. On one hand, the limited internal space of the artificial climate chamber may have impacted the development of the embryo sac in plants. On the other hand, this discrepancy may be related to our sampling of the entire ovary structure for *OsBBM1* expression analysis. Elevated temperatures could compromise the specificity of the egg cell-specific promoter, which may partly explain the increased expression observed after 35°C treatment. These findings suggest that the expression level of *OsBBM1* is one of the primary factors contributing to the transgenerational variation in induction rates observed in unstable lines.

The progressive decline in apomixis induction rates observed in line HW10 across generations likely results from transgene silencing, a phenomenon mediated through multiple molecular mechanisms: (1) Position effects, where suboptimal integration sites render transgenes susceptible to host genomic regulatory elements (Mathieu et al., 2003); (2) Homology-dependent silencing triggered by multicopy insertions that induce methylation-based repression (Mette et al., 2000); (3) Epigenetic regulation involving DNA methylation and histone modifications that alter chromatin accessibility (Law and Jacobsen, 2010); and (4) RNA-mediated silencing pathways where siRNA/miRNA molecules direct mRNA degradation or translational inhibition (Stroud et al., 2013).

In contrast to previous reports documenting stable inheritance patterns in apomictic materials (Khanday et al., 2019; Wang et al., 2019; Vernet et al., 2022; Wei et al., 2023; Song et al., 2024; Huang et al., 2025; Xiong et al., 2025; Song et al., 2024; Hu et al., 2025), our multi-generation and multi-location analysis of *OsBBM1*-mediated apomixis systems has for the first time revealed functional instability of the transgene insertion. This breakthrough finding necessitates comprehensive stability validation through extended multi-environmental generation trials in subsequent research, establishing a critical quality control standard for both fundamental studies and future commercial applications of apomictic rice. The demonstrated lability of exogenous *OsBBM1* expression underscores the imperative to incorporate generational stability monitoring as an essential component in apomixis-related breeding programs, fundamentally reshaping the evaluation framework for this transformative agricultural technology.

## Data Availability

The original contributions presented in the study are included in the article/**Supplementary Material**. Further inquiries can be directed to the corresponding authors.
