# Supplementary material for: High-frequency synthetic apomixis by OsBBM1 shows environmentally sensitive inheritance instability in hybrid rice
Source: Front Plant Sci. 2026 Feb 9;17:1747393. doi: 10.3389/fpls.2026.1747393 (PMC12926468; doi:10.3389/fpls.2026.1747393)
Supplement: Supplementary file 1 [file DataSheet1.docx]

Supplementary Material

##
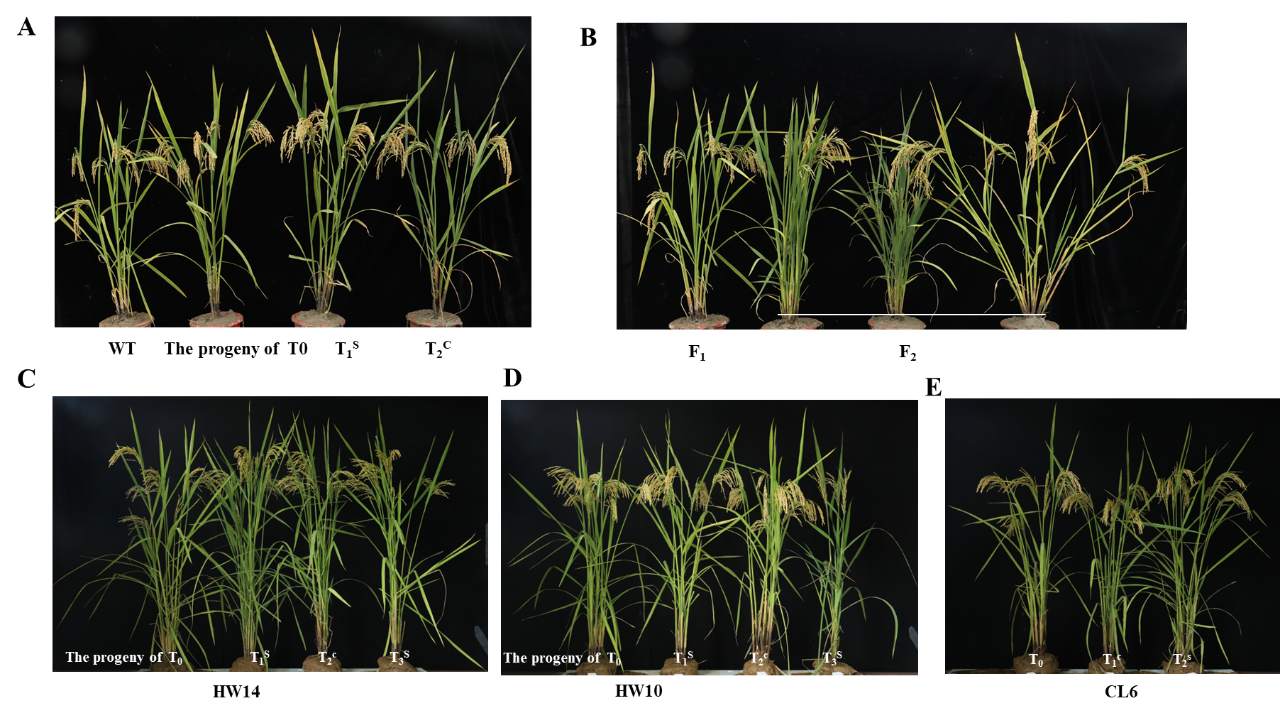
Supplementary Figures

**Supplementary Figure 1.** Analysis of clonal diploid plants and phenotypic characterization of apomictic materials cultivated

**A:** Plant phenotypes of p94C apomictic line HW7 across generations, plants arranged left to right represent the wild-type control Yongyou4949 (WT), and the progeny of T_0_, T_1_^s^, T_2_^c^ (T_1_, T_2_, T_3_). **B:** Plant phenotypes of wild-type control Yongyou4949 (F_1_ generation) and self-pollinated F_2_ progeny. **C-D:** Phenotypic performance of p94C apomictic lines HW14 and HW10 across generations, plants arranged left to right represent the progeny of T_0_, T_1_^s^, T_2_^c^, T_3_^s^. **E:** Phenotypic characteristics of p95C apomictic line CL6 across generations, plants arranged left to right represent the progeny of T_0_, T_1_^c^, T_2_^s^.

**Supplementary Tables**

Supplemental Table 1. Induction rates of five individual lines across the T_0_ to T_2_ generations of p94C and the T_1_ generation of p95C, cultivated in Sanya

| **construct** | **Line** | **generation** | Progeny tested | **diploid** | **%clonal seeds** |
| --- | --- | --- | --- | --- | --- |
| p94C | HW7 | T_0_ | 133 | 120* | 90.23 |
|  |  | T_1_^s^ | 211 | 211* | 100.00 |
|  |  | T_2_^c^ | 363 | 353* | 97.25 |
|  | HW10 | T_0_ | 26 | 22 | 84.62 |
|  |  | T_1_^s^ | 352 | 116* | 32.95 |
|  |  | T_2_^c^ | 438 | 27* | 6.16 |
|  | HW11 | T_0_ | 184 | 137* | 74.46 |
|  |  | T_1_^s^ | 299 | 266* | 88.96 |
|  |  | T_2_^c^ | 354 | 288* | 81.36 |
|  | HW14 | T_0_ | 78 | 68* | 87.18 |
|  |  | T_1_^s^ | 252 | 227* | 90.08 |
|  |  | T_2_^c^ | 463 | 84* | 18.14 |
|  | HW16 | T_0_ | 414 | 359* | 86.71 |
|  |  | T_1_^s^ | 410 | 379* | 92.44 |
|  |  | T_2_^c^ | 406 | 281* | 69.21 |
| p95C | CL6 | T_1_^c^ | 63 | 10 | 15.87 |
|  | CL10 | T_1_^c^ | 64 | 61 | 95.31 |
|  | CL11 | T_1_^c^ | 64 | 47 | 73.44 |
|  | CL12 | T_1_^c^ | 64 | 60 | 93.75 |
|  | CL13 | T_1_^c^ | 62 | 46 | 74.19 |

^S^ were grown at the Sanya transgenic base in Hainan; ^C^ were grown at Changsha transgenic base in Hunan, China; * involved the integration of flow cytometric ploidy analysis with field observations of agronomic traits.

Supplemental Table 2. Induction rates of five individual lines across the T_0_ to T_3_ generations of p94C and the T_0_ to T_2_ generation of p95C, cultivated in Changsha

| **Construct** | **Line** | **Generation** | Progeny tested | **Diploid** | **%clonal seeds** |
| --- | --- | --- | --- | --- | --- |
| p94C | HW7 | T_0_ | 35 | 32* | 91.43 |
|  |  | T_1_^s^ | 58 | 56* | 96.55 |
|  |  | T_2_^c^ | 48 | 45* | 93.75 |
|  |  | T_2_^s^ | 45 | 45* | 100.00 |
|  |  | T_3_^s^ | 33 | 33* | 100.00 |
|  | HW10 | T_0_ | 24 | 16* | 66.67 |
|  |  | T_1_^s^ | 56 | 22* | 39.29 |
|  |  | T_2_^c^ | 52 | 20* | 38.46 |
|  |  | T_2_^s^ | 43 | 10 | 23.26 |
|  |  | T_3_^s^ | 56 | 0 | 0.00 |
|  | HW11 | T_0_ | 31 | 20* | 64.52 |
|  |  | T_1_^s^ | 64 | 56* | 87.50 |
|  |  | T_2_^c^ | 46 | 32* | 69.57 |
|  |  | T_2_^s^ | 39 | 33* | 84.62 |
|  |  | T_3_^s^ | 47 | 43* | 91.49 |
|  | HW14 | T_0_ | 88 | 84* | 95.45 |
|  |  | T_1_^s^ | 35 | 33* | 94.29 |
|  |  | T_2_^c^ | 48 | 19 | 39.58 |
|  |  | T_2_^s^ | 67 | 56 | 83.58 |
|  |  | T_3_^s^ | 47 | 41 | 87.23 |
|  | HW16 | T_0_ | 72 | 64* | 88.89 |
|  |  | T_1_^s^ | 59 | 58 | 98.31 |
|  |  | T_2_^c^ | 55 | 45* | 81.82 |
|  |  | T_2_^s^ | 72 | 68* | 94.44 |
|  |  | T_3_^s^ | 49 | 44 | 89.80 |
| p95C | CL6 | T_0_ | 44 | 36 | 81.82 |
|  |  | T_1_^c^ | 51 | 11* | 21.57 |
|  |  | T_2_^s^ | 55 | 34* | 61.82 |
|  | CL10 | T_0_ | 32 | 29 | 90.63 |
|  |  | T_1_^c^ | 71 | 61* | 85.92 |
|  |  | T_2_^s^ | 59 | 59* | 100.00 |
|  | CL11 | T_0_ | 14 | 13 | 92.86 |
|  |  | T_1_^c^ | 60 | 45* | 75.00 |
|  |  | T_2_^s^ | 71 | 65 | 91.55 |
|  | CL12 | T_0_ | 38 | 37 | 97.37 |
|  |  | T_1_^c^ | 75 | 68* | 90.67 |
|  |  | T_2_^s^ | 56 | 55 | 98.21 |
|  | CL13 | T_0_ | 45 | 38* | 84.44 |
|  |  | T_1_^c^ | 47 | 36 | 76.60 |
|  |  | T_2_^s^ | 93 | 87 | 93.55 |

^S^ were grown at the Sanya transgenic base in Hainan; ^C^ were grown at Changsha transgenic base in Hunan, China；* involved the integration of flow cytometric ploidy analysis with field observations of agronomic traits.

Supplemental Table 3. Analysis of seed-setting rates in five individual lines across the T_0_ to T_2_ generations of p94C and the T_1_ generation of p95C, cultivated in Sanya

| **Construct** | **Line** | **Generation** | **Number** | **Actual grain** | **Total grain** | **Seed-setting rate%** |
| --- | --- | --- | --- | --- | --- | --- |
|  | YS | F_1_ | -1 | 1722 | 1816 | 94.82 |
|  |  |  | -2 | 1428 | 1502 | 95.07 |
|  |  |  | -3 | 1463 | 1551 | 94.33 |
|  |  |  | -4 | 1601 | 1715 | 93.35 |
|  |  |  | Average | 1554 | 1646 | 94.39 |
|  |  |  | SD | 135 | 145 | 0.76 |
| p94C | HW7 | T_1_ | -1 | 614 | 1766 | 34.77 |
|  |  |  | -2 | 1007 | 2222 | 45.32 |
|  |  |  | -3 | 657 | 1671 | 39.32 |
|  |  |  | -4 | 569 | 1561 | 36.45 |
|  |  |  | Average | 712 | 1805 | 38.96 |
|  |  |  | SD | 200 | 290 | 4.63 |
|  |  | T_2_ | -1 | 702 | 1915 | 36.66 |
|  |  |  | -2 | 544 | 1561 | 34.85 |
|  |  |  | -3 | 636 | 1758 | 36.18 |
|  |  |  | -4 | 767 | 2177 | 35.23 |
|  |  |  | Average | 662 | 1853 | 35.73 |
|  |  |  | SD | 95 | 260 | 0.83 |
|  |  | T_3_ | -1 | 652 | 1748 | 37.30 |
|  |  |  | -2 | 655 | 1928 | 33.97 |
|  |  |  | -3 | 442 | 1452 | 30.44 |
|  |  |  | -4 | 420 | 1345 | 31.23 |
|  |  |  | Average | 542 | 1618 | 33.24 |
|  |  |  | SD | 129 | 268 | 3.10 |
|  | HW10 | T_1_ | -1 | 1303 | 1868 | 69.75 |
|  |  |  | -2 | 1557 | 2930 | 53.14 |
|  |  |  | -3 | 1204 | 1973 | 61.02 |
|  |  |  | -4 | 1230 | 2069 | 59.45 |
|  |  |  | Average | 1324 | 2210 | 60.84 |
|  |  |  | SD | 161 | 487 | 6.85 |
|  |  | T_2_ | -1 | 1977 | 2704 | 73.11 |
|  |  |  | -2 | 1568 | 2270 | 69.07 |
|  |  |  | -3 | 1740 | 2419 | 71.93 |
|  |  |  | -4 | 1563 | 2552 | 61.25 |
|  |  |  | Average | 1712 | 2486 | 68.84 |
|  |  |  | SD | 195 | 185 | 5.34 |
|  |  | T_3_ | -1 | 1516 | 1987 | 76.30 |
|  |  |  | -2 | 1745 | 2113 | 82.58 |
|  |  |  | -3 | 1872 | 2406 | 77.81 |
|  |  |  | -4 | 1358 | 1660 | 81.81 |
|  |  |  | Average | 1623 | 2042 | 79.62 |
|  |  |  | SD | 230 | 309 | 3.05 |
|  | HW11 | T_1_ | -1 | 943 | 1296 | 72.76 |
|  |  |  | -2 | 957 | 1327 | 72.12 |
|  |  |  | -3 | 1570 | 2075 | 75.66 |
|  |  |  | -4 | 1588 | 2265 | 70.11 |
|  |  |  | Average | 1265 | 1741 | 72.66 |
|  |  |  | SD | 363 | 502 | 2.30 |
|  |  | T_2_ | -1 | 1573 | 2228 | 70.60 |
|  |  |  | -2 | 1498 | 2099 | 71.37 |
|  |  |  | -3 | 1509 | 2082 | 72.48 |
|  |  |  | -4 | 1453 | 2212 | 65.69 |
|  |  |  | Average | 1508 | 2155 | 70.03 |
|  |  |  | SD | 50 | 75 | 3.00 |
|  |  | T_3_ | -1 | 1170 | 1590 | 73.58 |
|  |  |  | -2 | 1332 | 1820 | 73.19 |
|  |  |  | -3 | 1155 | 1558 | 74.13 |
|  |  |  | -4 | 1479 | 2069 | 71.48 |
|  |  |  | Average | 1284 | 1759 | 73.10 |
|  |  |  | SD | 153 | 237 | 1.14 |
|  | HW14 | T_1_ | -1 | 904 | 1489 | 60.71 |
|  |  |  | -2 | 1164 | 2026 | 57.45 |
|  |  |  | -3 | 1188 | 1860 | 63.87 |
|  |  |  | -4 | 1284 | 2167 | 59.25 |
|  |  |  | Average | 1135 | 1886 | 60.32 |
|  |  |  | SD | 162 | 293 | 2.72 |
|  |  | T_2_ | -1 | 616 | 1510 | 40.79 |
|  |  |  | -2 | 884 | 2098 | 42.14 |
|  |  |  | -3 | 889 | 1818 | 48.90 |
|  |  |  | -4 | 682 | 1571 | 43.41 |
|  |  |  | Average | 768 | 1749 | 43.81 |
|  |  |  | SD | 140 | 268 | 3.56 |
|  |  | T_3_ | -1 | 890 | 1835 | 48.50 |
|  |  |  | -2 | 996 | 1862 | 53.49 |
|  |  |  | -3 | 551 | 1237 | 44.54 |
|  |  |  | -4 | 793 | 1719 | 46.13 |
|  |  |  | Average | 808 | 1663 | 48.17 |
|  |  |  | SD | 190 | 291 | 3.90 |
|  | HW16 | T_1_ | -1 | 1113 | 1568 | 70.98 |
|  |  |  | -2 | 1454 | 2080 | 69.90 |
|  |  |  | -3 | 1364 | 1818 | 75.03 |
|  |  |  | -4 | 1632 | 2234 | 73.05 |
|  |  |  | Average | 1391 | 1925 | 72.24 |
|  |  |  | SD | 216 | 293 | 2.27 |
|  |  | T_2_ | -1 | 891 | 1773 | 50.25 |
|  |  |  | -2 | 1353 | 1976 | 68.47 |
|  |  |  | -3 | 702 | 1132 | 62.01 |
|  |  |  | -4 | 649 | 996 | 65.16 |
|  |  |  | Average | 899 | 1469 | 61.48 |
|  |  |  | SD | 320 | 478 | 7.93 |
|  |  | T_3_ | -1 | 1771 | 2403 | 73.70 |
|  |  |  | -2 | 1267 | 1742 | 72.73 |
|  |  |  | -3 | 1078 | 1594 | 67.63 |
|  |  |  | -4 | 1024 | 1426 | 71.81 |
|  |  |  | Average | 1285 | 1791 | 71.47 |
|  |  |  | SD | 340 | 428 | 2.67 |
| p95C | CL6 | T_2_ | -1 | 370 | 968 | 38.22 |
|  |  |  | -2 | 693 | 1570 | 44.14 |
|  |  |  | -3 | 885 | 1841 | 48.07 |
|  |  |  | -4 | 729 | 1272 | 57.31 |
|  |  |  | -5 | 1283 | 2446 | 52.45 |
|  |  |  | Average | 792 | 1619 | 48.04 |
|  |  |  | SD | 332 | 566 | 7.37 |
|  | CL10 | T_2_ | -1 | 482 | 1483 | 32.50 |
|  |  |  | -2 | 872 | 2616 | 33.33 |
|  |  |  | -3 | 529 | 1594 | 33.19 |
|  |  |  | -4 | 554 | 1711 | 32.38 |
|  |  |  | -5 | 594 | 1975 | 30.08 |
|  |  |  | Average | 606 | 1876 | 32.30 |
|  |  |  | SD | 154 | 452 | 1.31 |
|  | CL11 | T_2_ | -1 | 816 | 1401 | 58.24 |
|  |  |  | -2 | 681 | 1091 | 62.42 |
|  |  |  | -3 | 931 | 1573 | 59.19 |
|  |  |  | -4 | 651 | 1105 | 58.91 |
|  |  |  | -5 | 629 | 1240 | 50.73 |
|  |  |  | Average | 742 | 1282 | 57.90 |
|  |  |  | SD | 128 | 205 | 4.32 |
|  | CL12 | T_2_ | -1 | 313 | 1166 | 26.84 |
|  |  |  | -2 | 375 | 1466 | 25.58 |
|  |  |  | -3 | 401 | 1303 | 30.78 |
|  |  |  | -4 | 338 | 1041 | 32.47 |
|  |  |  | -5 | 489 | 1898 | 25.76 |
|  |  |  | Average | 383 | 1375 | 28.29 |
|  |  |  | SD | 68 | 333 | 3.14 |
|  | CL13 | T_2_ | -1 | 479 | 993 | 48.24 |
|  |  |  | -2 | 652 | 1228 | 53.09 |
|  |  |  | -3 | 817 | 1303 | 62.70 |
|  |  |  | -4 | 832 | 1576 | 52.79 |
|  |  |  | -5 | 880 | 1469 | 59.90 |
|  |  |  | Average | 732 | 1314 | 55.35 |
|  |  |  | SD | 165 | 225 | 5.85 |

Supplemental Table 4. Analysis of seed-setting rates in five individual lines across the T_0_ to T_3_ generations of p94C and the T_0_ to T_2_ generation of p95C, cultivated in Changsha

| **Construct** | **Line** | **Generation** | **Number** | **Actual grain** | **Total grain** | **Seed-setting rate %** |
| --- | --- | --- | --- | --- | --- | --- |
|  | YS | **F_1_** | -1 | 3104 | 3788 | 81.94 |
|  |  |  | -2 | 2518 | 3148 | 79.99 |
|  |  |  | -3 | 2604 | 2972 | 87.62 |
|  |  |  | -4 | 2399 | 2768 | 86.67 |
|  |  |  | Average | 2656 | 3169 | 84.05 |
|  |  |  | SD | 310 | 441 | 3.68 |
| p94C | HW7 | T_1_ | -1 | 1272 | 3097 | 41.07 |
|  |  |  | -2 | 777 | 2014 | 38.58 |
|  |  |  | -3 | 1877 | 4766 | 39.38 |
|  |  |  | -4 | 810 | 1936 | 41.84 |
|  |  |  | Average | 1184 | 2953 | 40.22 |
|  |  |  | SD | 514 | 1320 | 1.50 |
|  |  | T_2_ | -1 | 971 | 3756 | 25.85 |
|  |  |  | -2 | 593 | 2474 | 23.97 |
|  |  |  | -3 | 1076 | 3697 | 29.10 |
|  |  |  | -4 | 745 | 2631 | 28.32 |
|  |  |  | Average | 846 | 3140 | 26.81 |
|  |  |  | SD | 218 | 681 | 2.35 |
|  |  | T_3_ | -1 | 617 | 2065 | 29.88 |
|  |  |  | -2 | 876 | 2574 | 34.03 |
|  |  |  | -3 | 626 | 2317 | 27.02 |
|  |  |  | -4 | 849 | 2454 | 34.60 |
|  |  |  | Average | 742 | 2353 | 31.38 |
|  |  |  | SD | 140 | 219 | 3.59 |
|  |  | T_4_ | -1 | 1078 | 3157 | 34.15 |
|  |  |  | -2 | 948 | 3367 | 28.16 |
|  |  |  | -3 | 686 | 2073 | 33.09 |
|  |  |  | -4 | 932 | 2727 | 34.18 |
|  |  |  | Average | 911 | 2831 | 32.39 |
|  |  |  | SD | 164 | 571 | 2.87 |
|  | HW10 | T_1_ | -1 | 1663 | 2655 | 62.64 |
|  |  |  | -2 | 2560 | 4036 | 63.43 |
|  |  |  | -3 | 1603 | 2617 | 61.25 |
|  |  |  | -4 | 2297 | 3885 | 59.12 |
|  |  |  | Average | 2031 | 3298 | 61.61 |
|  |  |  | SD | 472 | 767 | 1.89 |
|  |  | T_2_ | -1 | 2074 | 3163 | 65.57 |
|  |  |  | -2 | 1565 | 2358 | 66.37 |
|  |  |  | -3 | 1069 | 2948 | 36.26 |
|  |  |  | -4 | 2052 | 3914 | 52.43 |
|  |  |  | Average | 1690 | 3096 | 55.16 |
|  |  |  | SD | 476 | 643 | 14.13 |
|  |  | T_3_ | -1 | 2453 | 3734 | 65.69 |
|  |  |  | -2 | 1034 | 1492 | 69.30 |
|  |  |  | -3 | 1521 | 2321 | 65.53 |
|  |  |  | -4 | 1074 | 1715 | 62.62 |
|  |  |  | Average | 1521 | 2316 | 65.79 |
|  |  |  | SD | 660 | 1008 | 2.74 |
|  | HW11 | T_1_ | -1 | 947 | 2298 | 41.21 |
|  |  |  | -2 | 1776 | 3570 | 49.75 |
|  |  |  | -3 | 2592 | 4803 | 53.97 |
|  |  |  | -4 | 1125 | 2251 | 49.98 |
|  |  |  | Average | 1610 | 3231 | 48.73 |
|  |  |  | SD | 745 | 1213 | 5.37 |
|  |  | T_2_ | -1 | 1938 | 3881 | 49.94 |
|  |  |  | -2 | 1312 | 2568 | 51.09 |
|  |  |  | -3 | 1451 | 2788 | 52.04 |
|  |  |  | -4 | 861 | 1686 | 51.07 |
|  |  |  | Average | 1391 | 2731 | 51.03 |
|  |  |  | SD | 443 | 903 | 0.86 |
|  |  | T_3_ | -1 | 864 | 1661 | 52.02 |
|  |  |  | -2 | 1072 | 2180 | 49.17 |
|  |  |  | -3 | 2096 | 4050 | 51.75 |
|  |  |  | -4 | 1238 | 2406 | 51.45 |
|  |  |  | Average | 1318 | 2574 | 51.10 |
|  |  |  | SD | 541 | 1032 | 1.30 |
|  |  | T_4_ | -1 | 2113 | 4036 | 52.35 |
|  |  |  | -2 | 1155 | 2175 | 53.10 |
|  |  |  | -3 | 1272 | 2333 | 54.52 |
|  |  |  | -4 | 1639 | 3114 | 52.63 |
|  |  |  | Average | 1545 | 2915 | 53.15 |
|  |  |  | SD | 431 | 853 | 0.96 |
|  | HW14 | T_1_ | -1 | 2349 | 4152 | 56.58 |
|  |  |  | -2 | 2661 | 4668 | 57.01 |
|  |  |  | -3 | 1318 | 2279 | 57.83 |
|  |  |  | -4 | 1558 | 2793 | 55.78 |
|  |  |  | Average | 1972 | 3473 | 56.80 |
|  |  |  | SD | 637 | 1122 | 0.86 |
|  |  | T_2_ | -1 | 2409 | 4263 | 56.51 |
|  |  |  | -2 | 1817 | 3483 | 52.17 |
|  |  |  | -3 | 1929 | 3314 | 58.21 |
|  |  |  | -4 | 3192 | 5307 | 60.15 |
|  |  |  | Average | 2337 | 4092 | 56.76 |
|  |  |  | SD | 625 | 910 | 3.40 |
|  |  | T_3_ | -1 | 1612 | 3368 | 47.86 |
|  |  |  | -2 | 1090 | 2414 | 45.15 |
|  |  |  | -3 | 1479 | 2926 | 50.55 |
|  |  |  | -4 | 1048 | 2458 | 42.64 |
|  |  |  | Average | 1307 | 2792 | 46.55 |
|  |  |  | SD | 281 | 449 | 3.41 |
|  |  | T_4_ | -1 | 3422 | 5775 | 59.26 |
|  |  |  | -2 | 1624 | 3182 | 51.04 |
|  |  |  | -3 | 1644 | 3284 | 50.06 |
|  |  |  | -4 | 956 | 1930 | 49.53 |
|  |  |  | Average | 1912 | 3543 | 52.47 |
|  |  |  | SD | 1057 | 1610 | 4.57 |
|  | HW16 | T_1_ | -1 | 1145 | 2548 | 44.94 |
|  |  |  | -2 | 940 | 1748 | 53.78 |
|  |  |  | -3 | 1761 | 3344 | 52.66 |
|  |  |  | -4 | 1428 | 3300 | 43.27 |
|  |  |  | Average | 1319 | 2735 | 48.66 |
|  |  |  | SD | 356 | 753 | 5.32 |
|  |  | T_2_ | -1 | 1497 | 3812 | 39.27 |
|  |  |  | -2 | 1264 | 2567 | 49.24 |
|  |  |  | -3 | 1090 | 2755 | 39.56 |
|  |  |  | -4 | 866 | 1812 | 47.79 |
|  |  |  | Average | 1179 | 2737 | 43.97 |
|  |  |  | SD | 267 | 825 | 5.29 |
|  |  | T_3_ | -1 | 1082 | 1812 | 59.71 |
|  |  |  | -2 | 877 | 2126 | 41.25 |
|  |  |  | -3 | 644 | 1686 | 38.20 |
|  |  |  | -4 | 1308 | 2762 | 47.36 |
|  |  |  | Average | 978 | 2097 | 46.63 |
|  |  |  | SD | 284 | 481 | 9.52 |
|  |  | T_4_ | -1 | 1140 | 2228 | 51.17 |
|  |  |  | -2 | 1651 | 3350 | 49.28 |
|  |  |  | -3 | 1123 | 2245 | 50.02 |
|  |  |  | -4 | 1442 | 2408 | 59.88 |
|  |  |  | Average | 1339 | 2558 | 52.59 |
|  |  |  | SD | 254 | 534 | 4.92 |
| p95C | CL6 | T_1_ | -1 | 1265 | 3108 | 40.70 |
|  |  |  | -2 | 1081 | 2717 | 39.79 |
|  |  |  | -3 | 1831 | 4458 | 41.07 |
|  |  |  | -4 | 907 | 1941 | 46.73 |
|  |  |  | Average | 1271 | 3056 | 42.07 |
|  |  |  | SD | 401 | 1053 | 3.15 |
|  |  | T_2_ | -1 | 1383 | 3251 | 42.54 |
|  |  |  | -2 | 1419 | 3601 | 39.41 |
|  |  |  | -3 | 1430 | 3729 | 38.35 |
|  |  |  | -4 | 997 | 2178 | 45.78 |
|  |  |  | Average | 1307 | 3190 | 41.52 |
|  |  |  | SD | 208 | 704 | 3.35 |
|  |  | T_3_ | -1 | 2098 | 3788 | 55.39 |
|  |  |  | -2 | 1351 | 2706 | 49.93 |
|  |  |  | -3 | 1640 | 2843 | 57.69 |
|  |  |  | -4 | 1330 | 3195 | 41.63 |
|  |  |  | Average | 1605 | 3133 | 51.16 |
|  |  |  | SD | 358 | 483 | 7.14 |
|  | CL10 | T_1_ | -1 | 642 | 3469 | 18.51 |
|  |  |  | -2 | 639 | 2885 | 22.15 |
|  |  |  | -3 | 504 | 2281 | 22.10 |
|  |  |  | -4 | 438 | 2014 | 21.75 |
|  |  |  | Average | 556 | 2662 | 21.12 |
|  |  |  | SD | 102 | 650 | 1.75 |
|  |  | T_2_ | -1 | 936 | 3702 | 25.28 |
|  |  |  | -2 | 732 | 3075 | 23.80 |
|  |  |  | -3 | 534 | 2301 | 23.21 |
|  |  |  | -4 | 733 | 2953 | 24.82 |
|  |  |  | Average | 734 | 3008 | 24.28 |
|  |  |  | SD | 164 | 574 | 0.94 |
|  |  | T_3_ | -1 | 499 | 1921 | 25.98 |
|  |  |  | -2 | 600 | 1954 | 30.71 |
|  |  |  | -3 | 561 | 1897 | 29.57 |
|  |  |  | -4 | 550 | 2064 | 26.65 |
|  |  |  | Average | 553 | 1959 | 28.23 |
|  |  |  | SD | 42 | 74 | 2.27 |
|  | CL11 | T_1_ | -1 | 760 | 3341 | 22.75 |
|  |  |  | -2 | 479 | 1914 | 25.03 |
|  |  |  | -3 | 311 | 1634 | 19.03 |
|  |  |  | Average | 517 | 2296 | 22.27 |
|  |  |  | SD | 227 | 915 | 3.03 |
|  |  | T_2_ | -1 | 200 | 1121 | 17.84 |
|  |  |  | -2 | 418 | 2117 | 19.74 |
|  |  |  | -3 | 751 | 2982 | 25.18 |
|  |  |  | -4 | 411 | 2008 | 20.47 |
|  |  |  | Average | 445 | 2057 | 20.81 |
|  |  |  | SD | 228 | 761 | 3.12 |
|  |  | T_3_ | -1 | 274 | 1511 | 18.13 |
|  |  |  | -2 | 225 | 889 | 25.31 |
|  |  |  | -3 | 635 | 2112 | 30.07 |
|  |  |  | -4 | 491 | 2360 | 20.81 |
|  |  |  | Average | 406 | 1718 | 23.58 |
|  |  |  | SD | 191 | 658 | 5.24 |
|  | CL12 | T_1_ | -1 | 604 | 2669 | 22.63 |
|  |  |  | -2 | 410 | 2250 | 18.22 |
|  |  |  | -3 | 679 | 3222 | 21.07 |
|  |  |  | -4 | 476 | 2331 | 20.42 |
|  |  |  | Average | 542 | 2618 | 20.59 |
|  |  |  | SD | 122 | 442 | 1.83 |
|  |  | T_2_ | -1 | 433 | 2083 | 20.79 |
|  |  |  | -2 | 689 | 3222 | 21.38 |
|  |  |  | -3 | 507 | 2452 | 20.68 |
|  |  |  | -4 | 527 | 2558 | 20.60 |
|  |  |  | Average | 539 | 2579 | 20.86 |
|  |  |  | SD | 108 | 475 | 0.36 |
|  |  | T_3_ | -1 | 675 | 3171 | 21.29 |
|  |  |  | -2 | 524 | 2318 | 22.61 |
|  |  |  | -3 | 797 | 3652 | 21.82 |
|  |  |  | -4 | 432 | 1820 | 23.74 |
|  |  |  | Average | 607 | 2740 | 22.36 |
|  |  |  | SD | 161 | 825 | 1.06 |
|  | CL13 | T_1_ | -1 | 746 | 2217 | 33.65 |
|  |  |  | -2 | 1032 | 3026 | 34.10 |
|  |  |  | -3 | 967 | 2638 | 36.66 |
|  |  |  | -4 | 758 | 2139 | 35.44 |
|  |  |  | Average | 876 | 2505 | 34.96 |
|  |  |  | SD | 145 | 411 | 1.36 |
|  |  | T_2_ | -1 | 705 | 2928 | 24.08 |
|  |  |  | -2 | 800 | 2379 | 33.63 |
|  |  |  | -3 | 1232 | 3857 | 31.94 |
|  |  |  | -4 | 597 | 2306 | 25.89 |
|  |  |  | Average | 834 | 2868 | 28.88 |
|  |  |  | SD | 278 | 716 | 4.62 |
|  |  | T_3_ | -1 | 575 | 2045 | 28.12 |
|  |  |  | -2 | 799 | 2687 | 29.74 |
|  |  |  | -3 | 553 | 1603 | 34.50 |
|  |  |  | -4 | 761 | 2752 | 27.65 |
|  |  |  | Average | 672 | 2272 | 30.00 |
|  |  |  | SD | 126 | 548 | 3.13 |

Supplemental Table 5. Analysis of seed-setting rate of two individual lines from the T_3_ generation of p94C and p95C after treatment at 28°C and 35°C (Changsha)

| **Construct** | **Line** | **Generation** | **Temperature ℃** | **Number** | **Actual grain** | **Total grain** | **Seed-setting rate %** |
| --- | --- | --- | --- | --- | --- | --- | --- |
| p94C | HW7 | T_3_ | 28 | -1 | 85 | 728 | 11.68 |
|  |  |  |  | -2 | 70 | 703 | 9.96 |
|  |  |  |  | -3 | 75 | 729 | 10.29 |
|  |  |  | 35 | -1 | 21 | 709 | 2.96 |
|  |  |  |  | -2 | 24 | 725 | 3.31 |
|  |  |  |  | -3 | 19 | 714 | 2.66 |
|  | HW14 | T_3_ | 28 | -1 | 268 | 724 | 37.02 |
|  |  |  |  | -2 | 233 | 661 | 35.25 |
|  |  |  |  | -3 | 241 | 689 | 34.98 |
|  |  |  | 35 | -1 | 31 | 705 | 4.40 |
|  |  |  |  | -2 | 38 | 793 | 4.79 |
|  |  |  |  | -3 | 28 | 715 | 3.92 |
| p95C | CL6 | T_3_ | 28 | -1 | 204 | 614 | 33.22 |
|  |  |  |  | -2 | 218 | 713 | 30.58 |
|  |  |  |  | -3 | 228 | 706 | 32.29 |
|  |  |  | 35 | -1 | 24 | 636 | 3.77 |
|  |  |  |  | -2 | 28 | 727 | 3.85 |
|  |  |  |  | -3 | 32 | 786 | 4.07 |
|  | CL10 | T_3_ | 28 | -1 | 91 | 826 | 11.02 |
|  |  |  |  | -2 | 88 | 833 | 10.56 |
|  |  |  |  | -3 | 78 | 836 | 9.33 |
|  |  |  | 35 | -1 | 19 | 664 | 2.86 |
|  |  |  |  | -2 | 21 | 709 | 2.96 |
|  |  |  |  | -3 | 22 | 719 | 3.06 |
